# Detection of Mercury Ion with High Sensitivity and Selectivity Using a DNA/Graphene Oxide Hybrid Immobilized on Glass Slides

**DOI:** 10.3390/bios11090300

**Published:** 2021-08-27

**Authors:** Li Gao, Qiuxiang Lv, Ni Xia, Yuanwei Lin, Feng Lin, Bangxing Han

**Affiliations:** 1 School of Life Sciences, Jiangsu University, Zhenjiang 212013, China; 2211917020@stmail.ujs.edu.cn (Q.L.); 18352864165@163.com (N.X.); yuanweilin@pku.edu.cn (Y.L.); 2 Key Laboratory of Healthy Freshwater Aquaculture, Ministry of Agriculture, Zhejiang Institute of Freshwater Fisheries, Huzhou 313001, China; wwlinfeng@163.com; 3 College of Biological and Pharmaceutical Engineering, West Anhui University, Moon Island, Lu’an 237012, China

**Keywords:** mercury ion detection, graphene oxide, DNA, Dendrobium huoshanense

## Abstract

Excessive mercury ions (Hg^2+^) cause great pollution to soil/water and pose a major threat to human health. The high sensitivity and high selectivity in the Hg^2+^ detection demonstrated herein are significant for the research areas of analytical chemistry, chemical biology, physical chemistry, drug discovery, and clinical diagnosis. In this study, a series of simple, low-cost, and highly sensitive biochips based on a graphene oxide (GO)/DNA hybrid was developed. Hg^2+^ is detected with high sensitivity and selectivity by GO/DNA hybrid biochips immobilized on glass slides. The performance of the biosensors can be improved by introducing more phosphorothioate sites and complementary bases. The best limit of detection of the biochips is 0.38 nM with selectivity of over 10:1. This sensor was also used for Hg^2+^ detection in Dendrobium. The results show this biochip is promising for Hg^2+^ detection.

## 1. Introduction

Graphene oxide (GO) is derived from a new substance formed by the oxidation of graphene. It is mainly made by combining graphite with strong acids, such as KClO_3_, HNO_3_, KMnO_4_, and H_2_SO_4_ [1,2,3]. It contains hydrophilic oxygen-based groups, such as carboxyl groups and hydroxyl groups, giving it excellent hydrophilic activity and ensuring its ease of participation in various chemical reactions in aqueous solutions [4]. GO has a very high fluorophore quenching efficiency [5], which is suitable for use as a quenching material in sensors. Other chemical groups and DNA sequences [6,7,8] can also be reacted with and fixed on the GO surface to modify GO. The interaction between GO and DNA generally includes physical adsorption and chemical bonding. The physical adsorption process is quite simple, but the fixation rate is low [9]. DNA that is not physically adsorbed will cause background interference in the sensing system, resulting in excessive false positive signals. In contrast, the chemical ligation process is complicated, and the cost is high. However, the fixation rate of chemical bonding is satisfactory, and the unconnected DNA can be removed by other methods, such as centrifugation, which has strong anti-interference. The result of the biosensors based on the chemical bonding fixation of DNA sequence forms a stable sensing system, which can achieve fast, highly sensitive, and effective detection with high accuracy. In recent years, the application of GO in biosensing platforms has been a hot topic, and GO has become an indispensable experimental element in biosensor systems, among which the most common one is the application of GO in fluorescence sensors. Researchers often use nanomaterials containing GO and aptamers to perform highly sensitive detection of the target object, because the GO preparation process is quite simple with low cost and toxicity [10]. The detection limit of GO-based biosensors is considerably excellent due to the less experimental materials and equipment required in fluorescent-based sensors.

Due to its high toxicity and long-lasting bioaccumulation, excess heavy metal mercury ions can cause huge pollution to soil/water and are a major threat to human health, which has become a serious problem worldwide [11]. The American Environmental Protection Association (EPA) and the World Health Organization (WHO) limit the content of Hg^2+^ in drinking water to 1 μg/L [12], which means that it is important to develop biosensors with higher Hg^2+^ detecting sensitivity and selectivity. Once the heavy metal mercury ion enters the biological system, such as by eating contaminated food and drinking water, it will inhibit important processes inside the living body depending on the concentration. It may accumulate in many body organs, such as the liver, heart, kidney, and brain, and interfere with normal biological functions in various ways. In severe cases, it can cause heart, kidney, and brain diseases [13,14,15]. In addition, mercury ion is also found in crops irrigated with contaminated water; thereby, a method for Hg^2+^ detection in the environment urgently needs to be developed.

Recently, researchers are devoted to the development of fast, sensitive, and cost-effective portable biosensors for Hg^2+^, in which the methods generally include electrochemistry [16,17], field-effect transistors [18], surface plasmon resonance [19,20], Raman spectroscopy [21], colorimetric detection [22,23], and fluorescence [24]. Compared with traditional sensors, biochips can not only achieve high-throughput detection but also meet portability and efficiency [25], which have been developed to detect DNA [26], proteins/peptides [27], and heavy metal ions [28]. Specific to Hg^2+^ detection, Shi et al. [29] developed colorimetric biochips in microarrays. Through specially designed DNA hairpin strands as molecular recognition probes, combined with staining methods, detection and quantification of Hg^2+^ in various real samples can be simultaneously achieved. After the molecular probe is combined with Hg^2+^, the secondary structure of the hairpin is “forced” open, allowing the nanogold–streptavidin conjugate (or streptavidin–horseradish peroxidase conjugate) to attach to the biotin group and produce a visual signal when stained. Since the probe can be fixed at a specific position, the biochip can simultaneously recognize and quantify Hg^2+^. Jiang et al. [28] proposed an ultra-sensitive fluorescent probe (TR-Hg) for Hg^2+^ detection based on the mechanism of aggregation-induced emission (AIE) and dark through-bond energy transfer (DTBET), using tetrabenzene ethylene as a dark donor and rhodamine B thiolactone as an acceptor to form probes. By utilizing the advantages of DTBET to eliminate the emission leakage of dark donors and provide energy transfer efficiencies close to 100%, TR-Hg exhibits a fluorescence enhancement of more than 30,000 times after reacting with Hg^2+^. However, these methods have complicated procedures, many laboratory supplies and consumables, and high costs, which have limitations in practical applications. Herein, we propose a series of simple, low-cost, and highly sensitive biochips based on the GO/DNA hybrid. The biochip was prepared by NHS (N-Hydroxysuccinimide)\EDC (1-(3-Dimethylaminopropyl)-3-ethylcarbodiimide hydrochloride)-activated GO and DNA modified with phosphorothioate (PS) cleavage sites and Cy5 fluorophore, and the GO/DNA hybrid was further immobilized to glass slides to achieve highly sensitive and high-throughput detection of Hg^2+^. The best limit of detection (LOD) of the biochips achieves 0.38 nM with selectivity larger than 10:1.

## 2. Experimental Parts

### 2.1. Chemicals

The DNA sequences used in this study were all synthesized by Sangon Biotech (Shanghai, China) Co., Ltd. and purified by high-performance liquid chromatography (HPLC). After synthesis, the DNA sequences were confirmed by gas chromatography and mass spectrometry (GC-MS). The sail brand microscope slides (width 25.4 mm, length 76.2 mm, thickness 0.8 mm) were purchased from Nanjing Kangai Experimental Equipment Business Department. APTES (3-aminopropyltriethoxysilane) was purchased from Sigma-Aldrich (Shanghai, China) Co., Ltd. Analytically pure Hg(NO_3_)_2_ was purchased from Shandong Xiya Chemical Industry Co., Ltd. Analytically pure KCl, NaNO_3_, AgNO_3_, ZnCl_2_, MgCl_2_·6H_2_O, CaCl_2_·2H_2_O, CdCl_2_, Pb(CH_3_COO)_2_·3H_2_O, FeSO_4_·7H_2_O, CuSO_4_·5H_2_O, MnCl_2_·4H_2_O, PBS buffer, H_2_SO_4_, H_2_O_2_, absolute ethyl alcohol, and other reagents were obtained from Sinopharm Chemical Reagent (Shanghai, China) Co., Ltd.

### 2.2. Instruments

Fluorescence spectra were measured by CapitalBio Corporation’s LuxScan 10K microarray scanner. The scanner power, the photomultiplier tube (PMT), and the resolution were set to 100, 650, and 10 μm, respectively. Ultrapure water (18.2 MΩ·cm) was prepared by an UPH ultrapure water machine from Sichuan ULUPURE (Chengdu, China) Co., Ltd.

### 2.3. Preparation of DNA/GO Hybrid Biosensors

The slides were soaked in a mixed liquid of concentrated H_2_SO_4_ and H_2_O_2_ with a volume ratio of 7:3. After at least 2 h, the slides were washed with ultrapure water several times and dried. Then, slides were further rinsed with mixed 95% ethanol and 2~3% APTES in a total volume of 100 mL. After 30 min, the slides were then washed again with ultrapure water several times and dried to obtain amino group-treated slides. Then, 2.5 mL of a mixture containing 50 mM NHS and 200 mM EDC was added with 5 mL of 2 mg/mL GO and 2.5 mL of ultrapure water to react overnight at room temperature. It was centrifuged at 10,000 rpm for 30 min to remove the supernatant, and 5 mL of ultrapure water was then added to obtain activated GO with a concentration of 2 mg/mL. A total of 100 μL of the activated GO was added to the amino group-treated slide to react at room temperature for 16 h followed by aspirating the reaction liquid with a pipette and washing it with ultrapure water several times (remove the unreacted activated GO). After drying, 50 nM DNA with the modified PS cleavage site was added to the slides and left at 16 °C for 16 h in the dark; then, the reaction solution was aspirated with a pipette. The slides were washed several times with PBS buffer (remove unreacted DNA) and dried. Finally, different concentrations of Hg^2+^ were added to react with the slides at 37 °C for 30 min. After the reaction solution was aspirated with a pipette, the slides were washed several times with PBS buffer and then dried. The fluorescence intensity was obtained by scanning the slide through the CapitalBio Corporation’s LuxScanTM 10K microarray chip scanner.

## 3. Results and Discussion

### Detection of Hg^2+^ Based on GO Microarray

**Figure 1:**
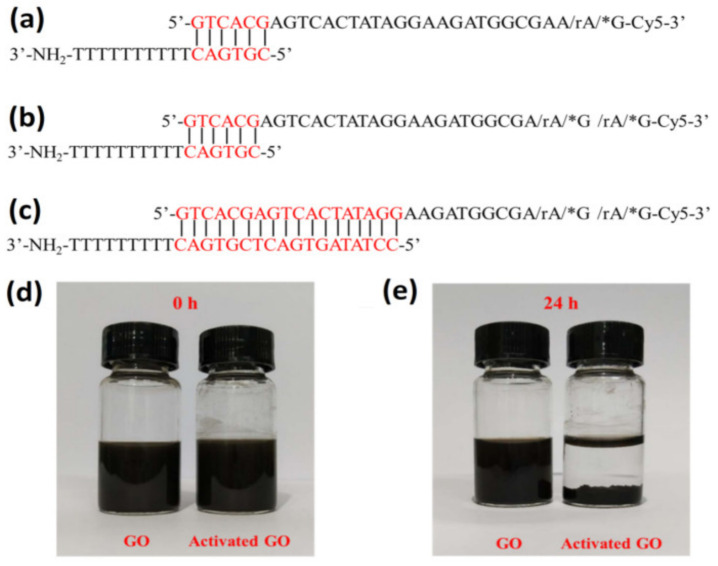
DNA sequence I hybrid with one PS cutting site (**a**) and two PS cutting sites (**b**). (**c**) DNA sequence II hybrid with two PS cutting sites. Digital images with comparison of GO and activated GO: (**d**) at the beginning and (**e**) after 24 h.

**Scheme 1:**
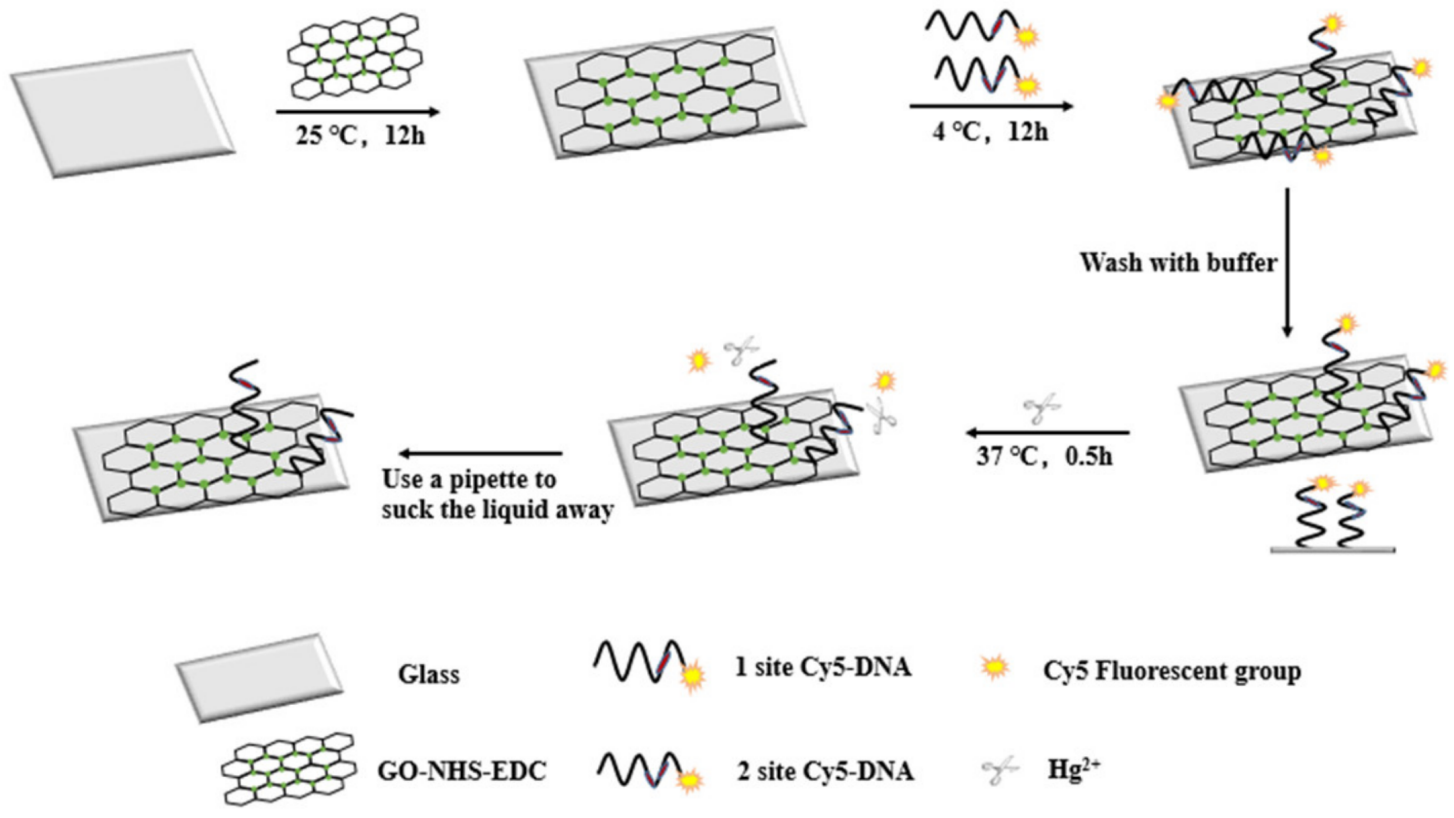
The mercury ions detection based on graphene oxide microarray.

The DNA sequences used in this study are presented in Figure 1a–c. As shown in Scheme 1, the hydroxyl group (-OH) was assembled on the slides with concentrated H_2_SO_4_ and H_2_O_2_, and the amino group (-NH_2_) was finally terminated after adding APTES to be combined with the exposed -OH on the slides. The reaction between -NH_2_ on the slides and the carboxyl group (-COOH) carried by GO is convenient for immobilizing the activated GO on the surface of the slides (NHS/EDC activates the carboxyl group of GO, Figure 1d,e). The -NH_2_-modified DNA was added to react with the activated GO, and the DNA was also immobilized on the surface of the slides. Finally, Hg^2+^ was added to perform PS cleavage on the DNA, and the Cy5 fluorescent group on the DNA was released into the reaction solution. After the reaction liquid was sucked away by the pipette gun, the fluorescence intensity of the slides decreased, according to the principle of fluorescence resonance energy transfer (FRET) [30,31]. With the increase in the concentration of Hg^2+^, the cutting rate will increase, and the fluorescence intensity of the corresponding slides will become lower. Thus, the quantitative detection of the analyte can be performed according to the change in the fluorescence intensity before and after adding the Hg^2+^.

**Figure 2:**
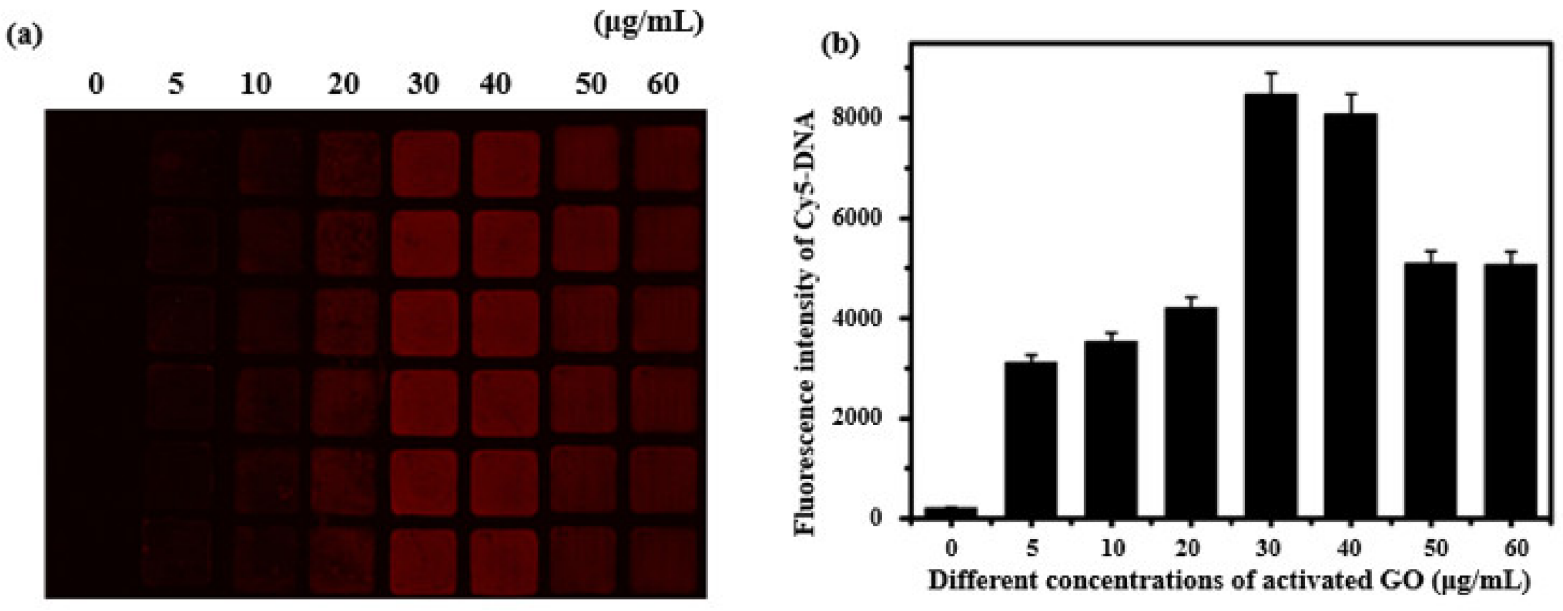
Optimization of activated GO concentration based on sensors containing DNA sequence I hybrid with one PS cutting site. (**a**) Scanning image of the chip microarray after optimization of activated GO concentration. (**b**) The fluorescence intensity of the Cy 5-DNA in the presence of different concentrations of activated GO.

DNA sequence I was first used to perform complementary hybridization with one PS site and two PS sites. After hybridization at 95 °C for 5 min, it was left at room temperature for 1~2 h. After adding different volumes of 2 mg/mL of activated GO to the treated slides with the final concentrations of 0, 5, 10, 20, 30, 40, 50, and 60 μg/mL, 50 nM of hybridized DNA at 4 °C was added. The reaction was protected from illumination for 16 h, and, finally, the concentration of activated GO was optimized by fixing the DNA concentration onto a slide. The fluorescence scanning results are shown in Figure 2a and Figure S1a. In both the two GO/DNA hybrid systems, the fluorescence intensity corresponding to 30 μg/mL of activated GO is the highest (Figure 2b and Figure S1b), indicating that the effect of immobilizing DNA is the best. Thus, 30 μg/mL of activated GO is selected as the optimal concentration for the following experiments.

**Figure 3:**
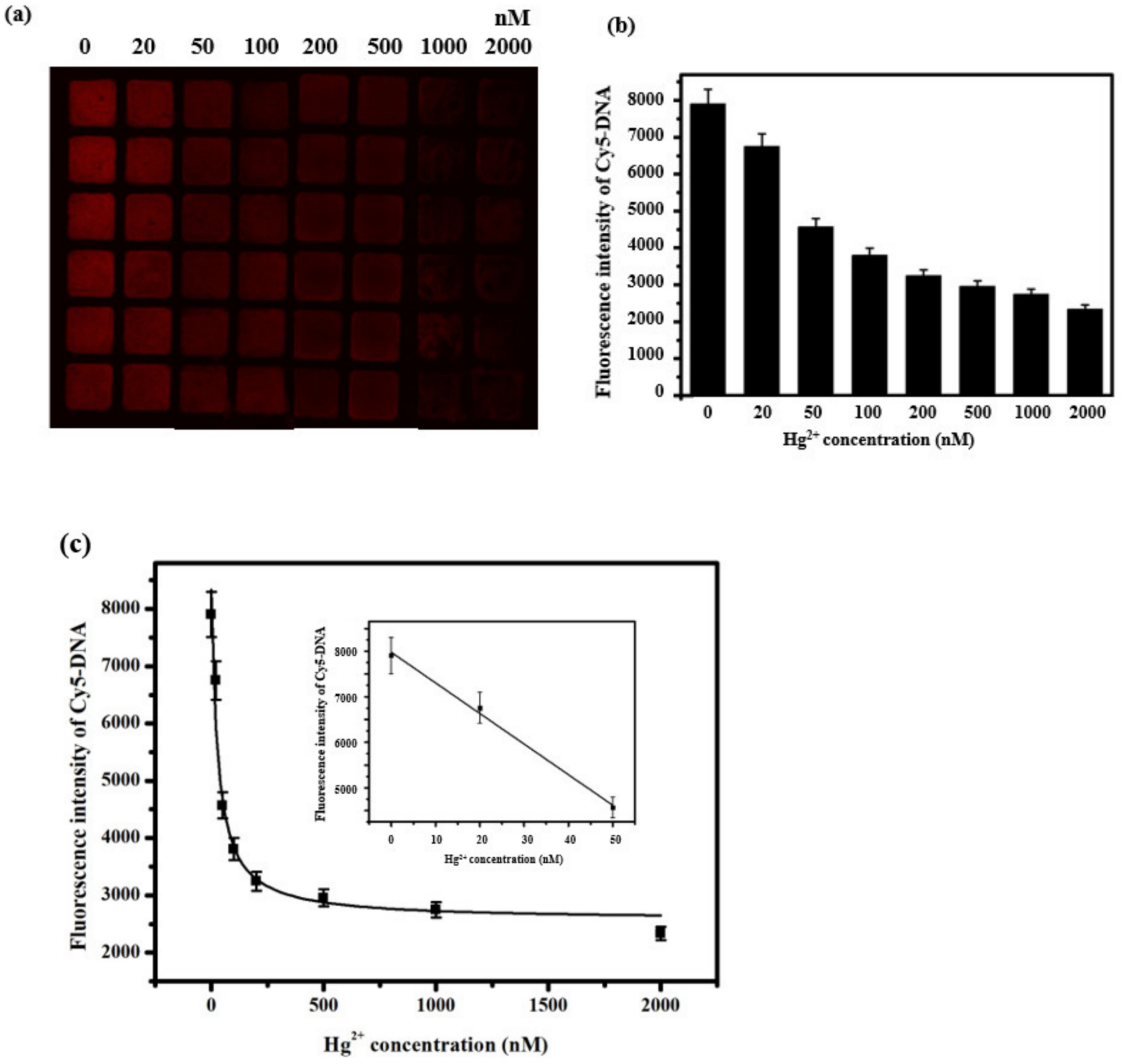
Sensitivity analysis for Hg^2+^ detection based on sensors containing DNA sequence I hybrid with one PS cutting site. (**a**) Scanning image of the chip array after adding different concentrations of Hg^2+^. (**b**)The fluorescence intensity of the Cy 5-DNA in the presence of different concentrations of Hg^2+^. (**c**) Plotted data extracted from (**b**). The inset shown in (**c**) represents the linear working curve with the average of 3 experiments at low mercury ion concentrations.

To obtain the concentration analysis working curve of Hg^2+^ detection, different concentrations of Hg^2+^ (0, 20, 50, 100, 200, 500, 1000, and 2000 nM) were added to the GO/DNA hybrid biochip. The slides were washed and dried after being left at 37 °C in the dark for 30 min. The fluorescence intensity caused by Hg^2+^ with different concentrations was measured (Figure 3a and Figure S2a). As the concentration of Hg^2+^ increases, the cleavage rate of PS DNA increases, and the fluorescence intensity of the remaining hybridized DNA immobilized on the slides becomes weaker. Compared with Figure 3b and Figure S2b, it can be observed that the number of PS cleavage sites can provide assistance to the cleavage rate, and the fluorescence intensity with more PS cleavage sites changes significantly. When the concentration of Hg^2+^ is 0~50 nM, the fluorescence intensity of the GO/DNA sequence I biochips with one PS site and two PS sites both decrease linearly with the increase in the concentration of Hg^2+^ (Figure 3c and Figure S2c). The linear equation corresponding to the GO/DNA sequence I biochip with one PS site is *y* = −67.14274*x* + 7974.83113 (*R*^2^ = 0.99236), with a LOD of 1.75 nM. Similarly, the linear equation corresponding to the GO/DNA sequence I biochip with two PS sites is *y* = −48.92359*x* + 8642.76321(*R*^2^ = 0.968) with a LOD of 1.49 nM.

In order to ensure the sensitivity and selectivity of the biochips, different metal ions, including Hg^2+^, were investigated and compared. After 1 μM of various metal ions (Cu^2+^, Mn^2+^, Pb^2+^, Fe^2+^, Zn^2+^, Ca^2+^, Mg^2+^, Ag^+^, K^+^, Na^+^, Cd^2+^, and Hg^2+^) were incubated to the DNA/GO hybrid biochips at 37 °C for 30 min, the slides were washed again and dried. The change in fluorescence intensity was further checked by the fluorescence scanner (Figure S3a,b). The fluorescence intensity corresponding to Hg^2+^ in both DNA/GO hybrid biochips is relatively weak, indicating that the biochips have good selectivity for Hg^2+^ detection, especially for the GO/DNA sequence I biochip with two PS sites. The decrease in fluorescence intensity (Δ*F*) after adding Hg^2+^ to the GO/DNA sequence I biochip with two PS sites is greater than that of the GO/DNA sequence I biochip with two PS sites, which demonstrates that increasing the PS cleavage site could enhance the selectivity.

Replacing DNA sequence I with DNA sequence II and hybridizing with two PS sites, DNA may further increase the sensitivity and selectivity of the biochip, because DNA sequence II has a higher complementary binding rate. Similar to the experiments conducted in DNA sequence I, the different concentrations of activated GO (0, 5, 10, 20, 30, 40, 50, and 60 μg/mL) in the GO/DNA sequence II biochip with two PS sites were investigated (Figure S4a). The fluorescence intensity corresponding to GO activated of 40 μg/mL is the highest (Figure S4b), indicating that the effect of DNA immobilization at this concentration is the best. GO is the optimal concentration for subsequent experiments. Thus, 40 μg/mL of activated GO is selected as the optimal concentration for the following experiments with DNA sequence II. Then, different concentrations of Hg^2+^ (0, 2, 5, 10, 20, 50, 100, 200, 500, and 1000 nM) were added to the GO/DNA hybrid biochip, and the changes in fluorescence intensity were measured by a fluorescence scanner (Figure S5a). As the Hg^2+^ concentration increases, the fluorescence intensity detected by the biochip becomes lower (Figure S5b). When the Hg^2+^ concentration is 0~10 nM, the fluorescence intensity of the GO/DNA sequence II biochip with two PS sites decreases linearly with the Hg^2+^ concentration increase (Figure S5c). The linear equation is *y* = 137.91347*x* + 5273.51532 (*R*^2^ = 0.97), and the LOD is 0.38 nM. Compared with that of DNA sequence I, the hybridization effect of DNA sequence II is more stable, and the LOD of the biochip based on DNA sequence II is better. As shown in Table 1 below, the detection limits of Hg^2+^ observed in this study are compared with the detection limits obtained by other assays. The sensor has a low detection limit.

**Figure 4:**
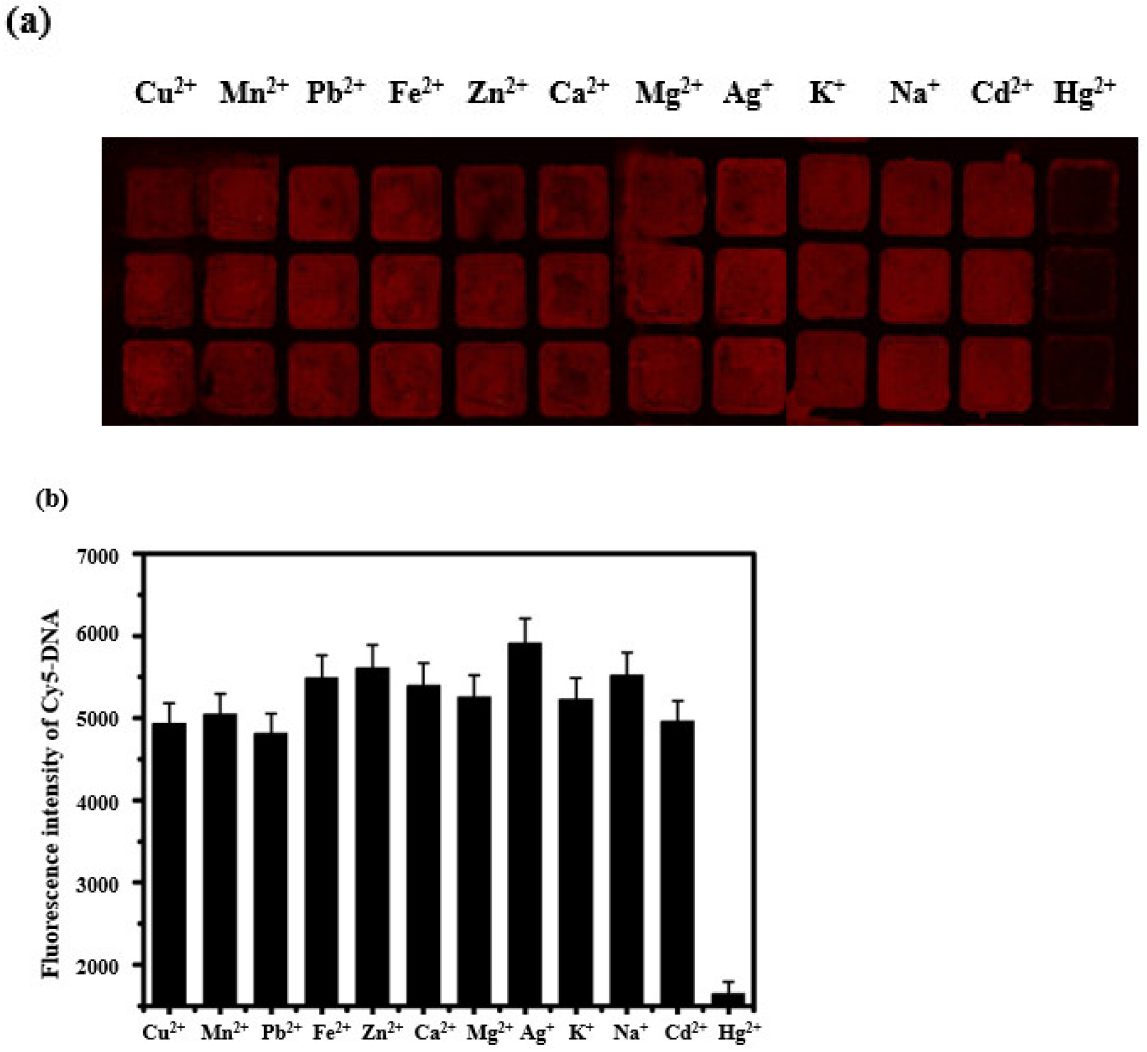
Selectivity analysis for Hg^2+^ detection based on sensors containing DNA sequence II hybrid with two PS cutting sites. (**a**) Scanning image of the chip microarray after adding various metal ions. (**b**) The fluorescence intensity of the Cy 5-DNA in the presence of different metal ions. Each concentration of the metal ion was fixed at 1 μM.

Similar to the selectivity exploration conducted in DNA sequence I, 1μM of various metal ions (Cu^2+^, Mn^2+^, Pb^2+^, Fe^2+^, Zn^2+^, Ca^2+^, Mg^2+^, Ag^+^, K^+^, Na^+^, Cd^2+^, and Hg^2+^) was incubated to the DNA/GO hybrid biochips with DNA sequence II. The change in fluorescence intensity was checked by the fluorescence scanner (Figure S4a). The fluorescence intensity corresponding to Hg^2+^ in the DNA/GO hybrid biochip is the weakest (over 10 times weaker than that of the other metal ions), which demonstrates that the selectivity of the biochip of the GO/DNA sequence II biochip with two PS sites has better selectivity for Hg^2+^ than that of DNA sequence I (Figure 4b and Figure S3d).

In addition, to further demonstrate the Hg^2+^ detection ability in real applications, some liquid samples collected from Dendrobium candidum and Dendrobium huoshanense grown in a natural environment were measured by the above-mentioned GO chip, and the results were negative (no obvious Hg^2+^ was observed). The GO chip can also be used to detect other targets, such as toxins, in Dendrobium. Therefore, it is an important tool for food safety, medical diagnosis, environmental monitoring, and other fields. 

## 4. Conclusions

In conclusion, three DNA/GO hybrid biochips were developed with high sensitivity and selectivity for Hg^2+^ detection. The chips were prepared based on the fixation of activated GO with an amino slide and contain two different DNA sequences with one or two PS sites. The LODs are 1.75 nM, 1.49 nM, and 0.38 nM. The sensitivity and selectivity can be improved by introducing more PS sites and complementary bases into the sequence. This biochip was used for Hg^2+^ detection in real applications, some liquid samples collected from Dendrobium. Though the real-time detection of the biochips needs to be further investigated in the future, the biochips demonstrated in this study have high sensitivity and selectivity with high throughput and portability, which have great potential application prospects in the detection of the heavy metal ion Hg^2+^. The results show this biochip is promising.

## Figures and Tables

**Table 1:** A comparison of our method with other methods for Hg^2+^ detection.

Method	Linear Range	Detection Limit (LOD)	References
Colorimetric	25–750 nM	50 nM	[32]
Colorimetric	25–40 nM	5 nM	[33]
Colorimetric	10 μM–1 mM	0.316 μM	[34]
Fluorescence	20–200 nM	20 nM	[35]
Fluorescence	20 nM–5 μM	12 nM	[36]
Fluorescence	10 nM–1.4 μM	4.6 nM	[37]
Fluorescence	0~10 nM	0.38 nM	This study

## Data Availability

Not applicable.

## Supplementary Materials

The following are available online at https://www.mdpi.com/article/10.3390/bios11090300/s1, Figure S1: Optimization of activated GO concentration based on sensors containing DNA sequences I hybrid with two PS cutting sites. Figure S2: Sensitivity analysis for Hg^2+^ detection based on sensors containing DNA sequences I hybrid with two PS cutting sites. Figure S3: Selectivity analysis for Hg^2+^ detection based on sensors containing DNA sequences I. Figure S4 Optimization of activated GO concentration based on sensors containing DNA sequences II hybrid with two PS cutting sites. Figure S5 Sensitivity analysis for Hg^2+^ detection based on sensors containing DNA sequences II hybrid with two PS cutting sites.
