# Supplementary material for: Detection of Mercury Ion with High Sensitivity and Selectivity Using a DNA/Graphene Oxide Hybrid Immobilized on Glass Slides
Source: Biosensors (Basel). 2021 Aug 27;11(9):300. doi: 10.3390/bios11090300 (PMC8471904; doi:10.3390/bios11090300)
Supplement: Supplementary file 1 [file biosensors-11-00300-s001.zip › biosensors-1339664-SI.pdf]

biosensors

MDPI

Supporting Information

Detection of Mercury Ion with High Sensitivity and Selectivity Using DNA/Graphene Oxide Hybrid Immobilized on Glass Slides

Li Gao [1,*], Qiuxiang Lv [1], Ni Xia [1], Yuanwei Lin [1], Feng Lin [2] and Bangxing Han [3,*]

[1] School of Life Sciences, Jiangsu University, Zhenjiang 212013, China; 2211917020@stmail.ujs.edu.cn (Q.L.); 18352864165@163.com (N.X.); yuanweilin@pku.edu.cn (Y.L.)

[2] Key Laboratory of Healthy Freshwater Aquaculture, Ministry of Agriculture, Zhejiang Institute of Freshwater Fisheries, Huzhou 313001, China; wwlinfeng@163.com

[3] College of Biological and Pharmaceutical Engineering, West Anhui University, Moon Island, Lu'an 237012, China

* Correspondence: gaoli@ujs.edu.cn (L.G.); hanbx1978@sina.com (B.H.)

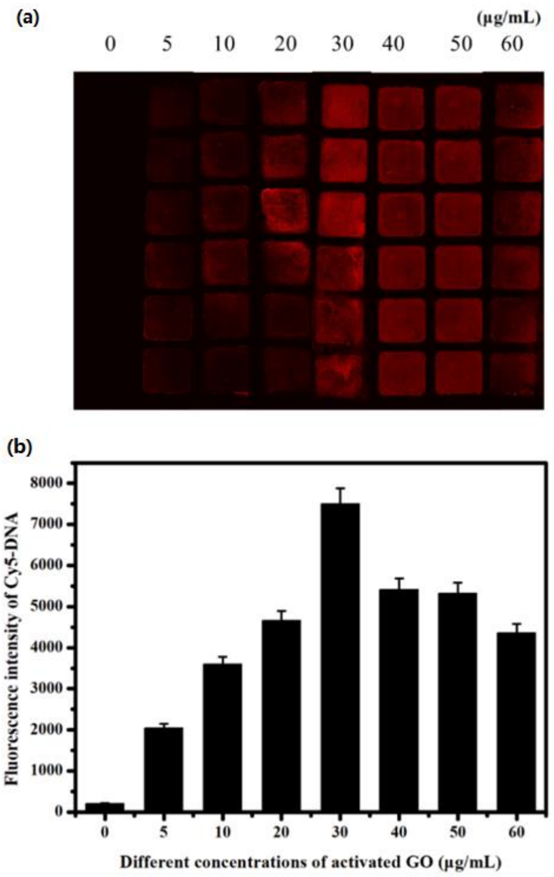

Figure S1 Optimization of activated GO concentration based on sensors containing DNA sequences I hybrid with two PS cutting sites. (**a**) Scanning image of the chip microarray after optimization of activated GO concentration; (**b**) The fluorescence intensity of the Cy 5-DNA in the presence of different concentrations of activated GO.

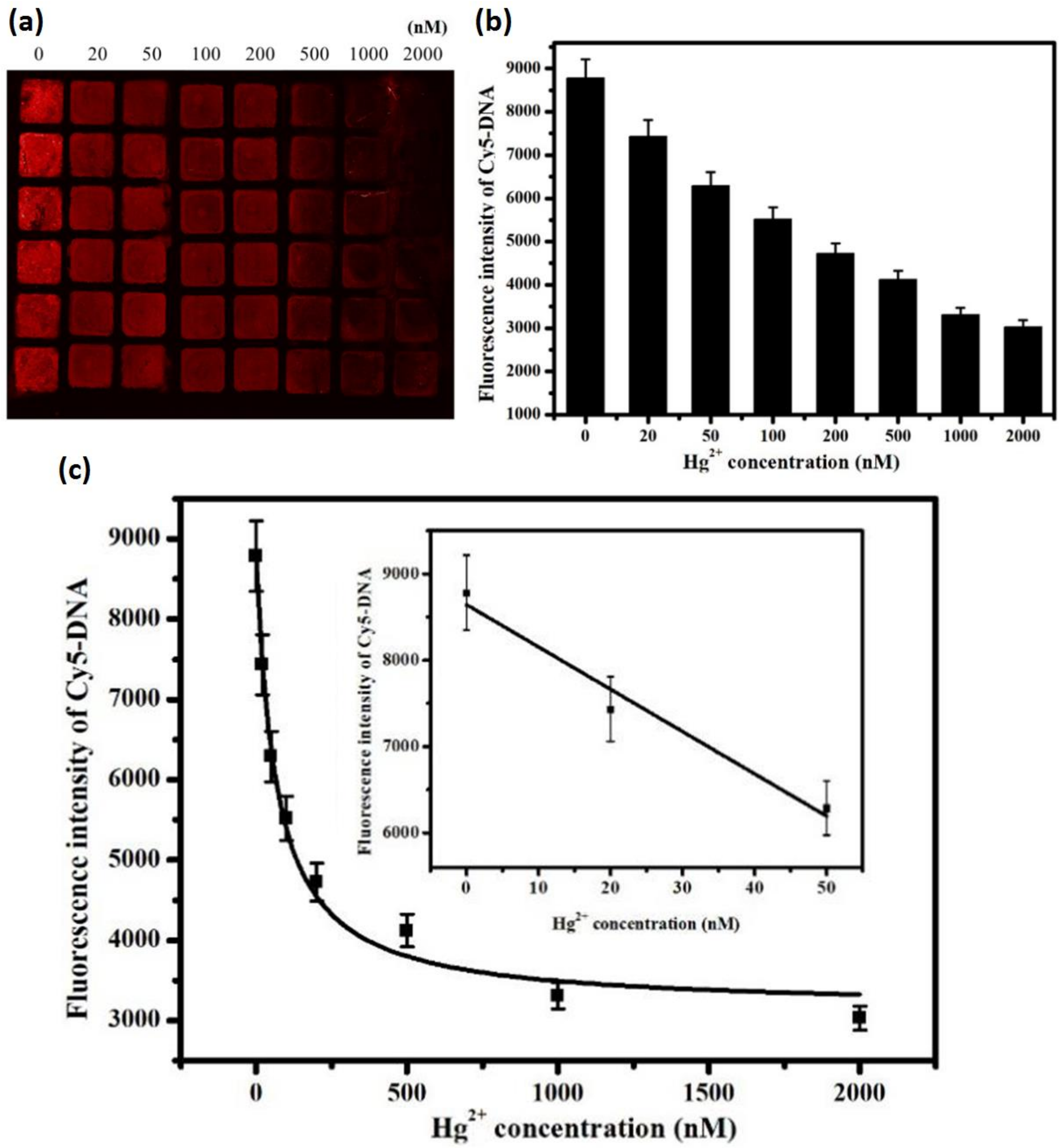

Figure S2 Sensitivity analysis for Hg^{2+} detection based on sensors containing DNA sequences I hybrid with two PS cutting sites. (**a**) Scanning image of the chip array after adding different concentrations of Hg^{2+}; (**b**) The fluorescence intensity of the Cy 5-DNA in the presence of different concentrations of Hg^{2+}; (**c**) Plotted data extracted from (b). The inset shown in (**c**) represents the linear working curve with the average of 3 experiments at low mercury ion concentrations.

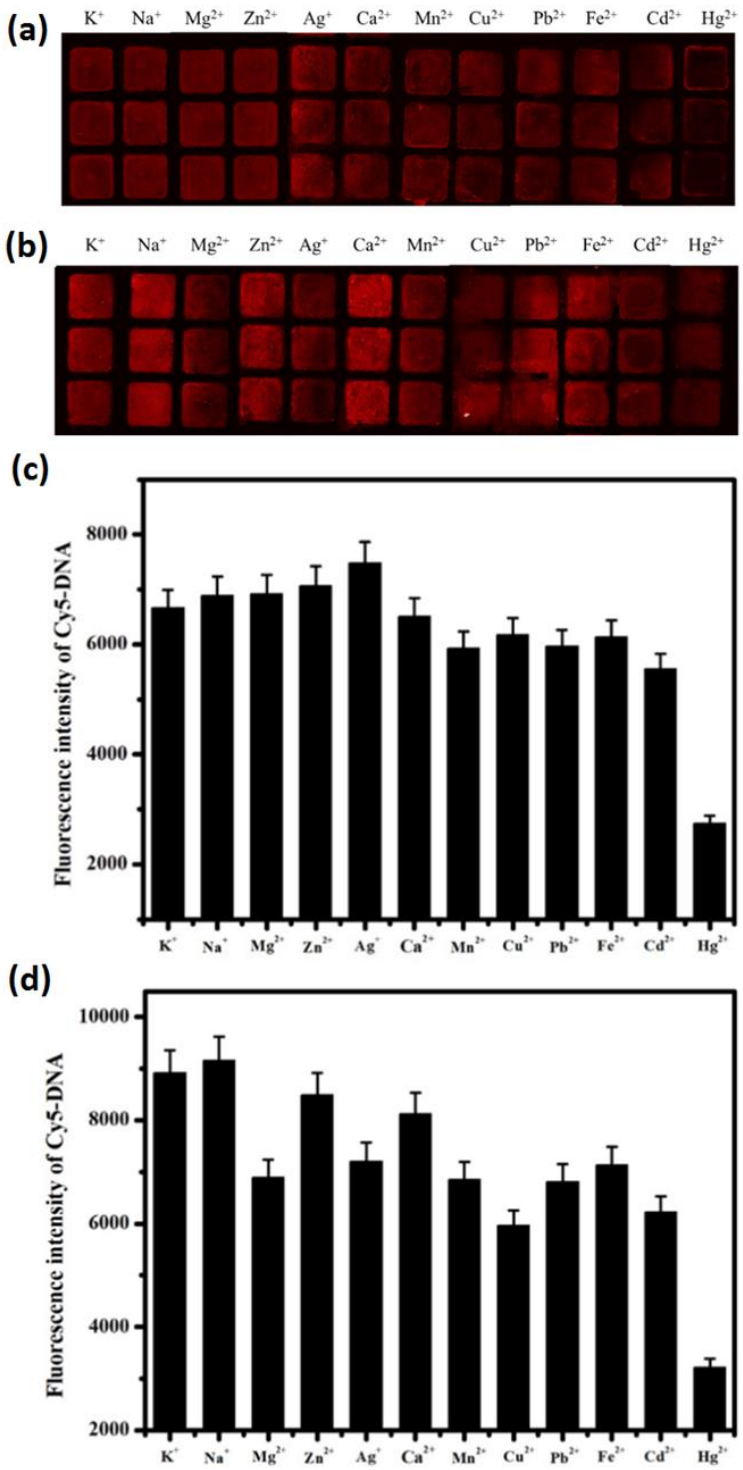

Figure S3 Selectivity analysis for Hg^{2+} detection based on sensors containing DNA sequences I. Scanning images of the chip microarrays after adding various metal ions: (**a**) hybrid with one PS cutting site; (**b**) hybrid with two PS cutting sites. The fluorescence intensity of the Cy 5-DNA in the presence of different metal ions: (**c**) hybrid with one PS cutting site; (**d**) hybrid with two PS cutting sites.Each concentration of the metal ion was fixed at 1μM.

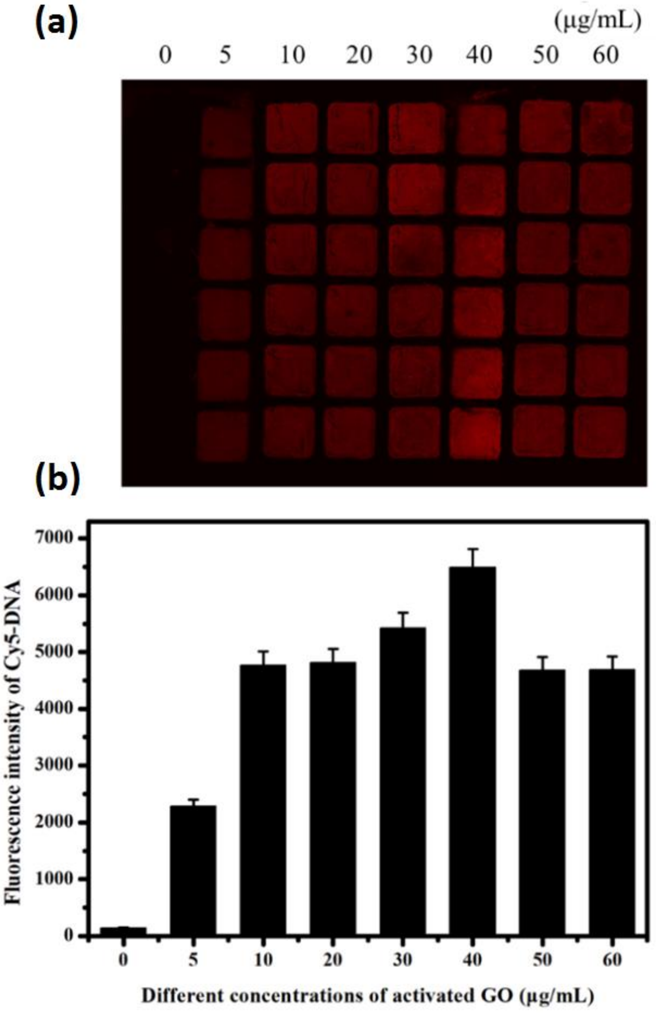

Figure S4 Optimization of activated GO concentration based on sensors containing DNA sequences II hybrid with two PS cutting sites. (**a**) Scanning image of the chip microarray after optimization of activated GO concentration; (**b**) The fluorescence intensity of the Cy 5-DNA in the presence of different concentrations of activated GO.

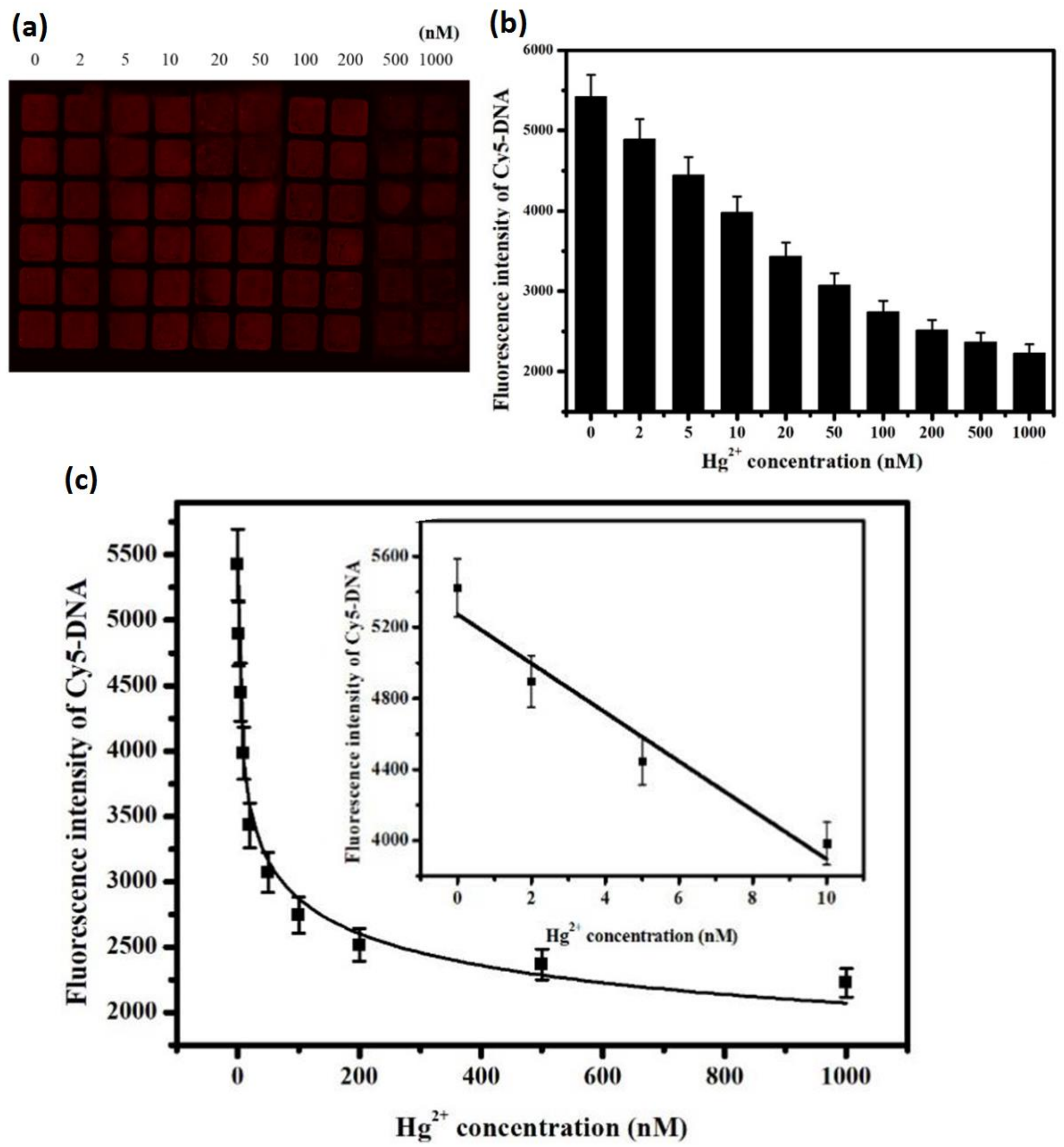

Figure S5 Sensitivity analysis for Hg^{2+} detection based on sensors containing DNA sequences II hybrid with two PS cutting sites. (**a**) Scanning image of the chip array after adding different concentrations of Hg^{2+}; (**b**) The fluorescence intensity of the Cy 5-DNA in the presence of different concentrations of Hg^{2+}; (**c**) Plotted data extracted from (**b**). The inset shown in (**c**) represents the linear working curve with the average of repeated 3 experiments at low mercury ion concentrations.
